# Corticosteroid Therapy in Optic Neuropathy Secondary to Nasopharyngeal Carcinoma

**DOI:** 10.7759/cureus.13735

**Published:** 2021-03-06

**Authors:** Zulaikha Wahab, Evelyn Tai, Wan-Hazabbah Wan Hitam, Khairy Shamel Sonny Teo

**Affiliations:** 1 Department of Ophthalmology and Visual Sciences, School of Medicine Sciences, Health Campus, Universiti Sains Malaysia, Kubang Kerian, MYS; 2 Ophthalmology, Hospital Universiti Sains Malaysia, Kubang Kerian, MYS

**Keywords:** nasopharyngeal carcinoma, orbital invasion, optic neuropathy, corticosteroid therapy, infiltrative neuropathy

## Abstract

Introduction: Nasopharyngeal carcinoma (NPC) is a tumor arising from the epithelial cells of the nasopharynx. NPC can spread and invade the base of skull, nasal cavity, paranasal sinuses, pterygopalatine fossa, and apex of the orbit. However, the involvement of the optic nerve in NPC is rare. The purpose of this case report is to report the efficacy of corticosteroid therapy in optic neuropathy secondary to NPC.

Clinical case: A 56-year-old Chinese woman, an active smoker, presented with a hearing deficit, persistent tinnitus and nasal congestion. Examination and investigations revealed the presence of a mass in the nasopharynx. Tissue biopsy revealed nasopharyngeal carcinoma. However, the Epstein-Barr virus was not tested. She was counseled for chemotherapy, but refused and was subsequently lost to follow up. She presented one year later with right eye ptosis associated with progressive worsening of diplopia and blurring of vision. Examination revealed multiple (second, third, fourth and sixth) cranial nerve involvement. Systemic examination and investigations revealed cervical lymphadenopathy and liver metastasis. Repeated imaging showed that the mass had invaded the base of the skull, cavernous sinus and orbital apices. Pulse dosing of corticosteroid therapy was commenced, resulting in dramatic improvement of vision.

Conclusion: Optic neuropathy may be the presenting sign of NPC. Corticosteroid therapy can offer immediate visual improvement.

## Introduction

Nasopharyngeal carcinoma (NPC) is a tumor arising from the epithelial cells of the nasopharynx. Risk factors for NPC include genetic susceptibility, environmental factors, and infection with Epstein-Barr virus (EBV) [1]. Cranial nerve involvement occurs when the skull base is invaded by direct extension of the tumor [2]. NPC may also spread anteriorly, involving the nasal cavity, paranasal sinuses, pterygopalatine fossa, and apex of the orbit [3]. Optic nerve involvement in NPC is rare [4]. We present this case to illustrate the use of corticosteroid therapy in optic neuropathy secondary to NPC.

## Case presentation

A 56-year-old Chinese woman, an active smoker, presented with hearing deficit and persistent tinnitus for a few months, associated with nasal congestion. Nasal endoscopy identified a mass in the nasopharynx. Histopathological evaluation of a tissue biopsy revealed nasopharyngeal carcinoma (non-keratinizing squamous cell carcinoma, undifferentiated type with no evaluation of EBV). She was counselled for chemoradiotherapy, but refused, citing personal reasons.

**Figure 1:**
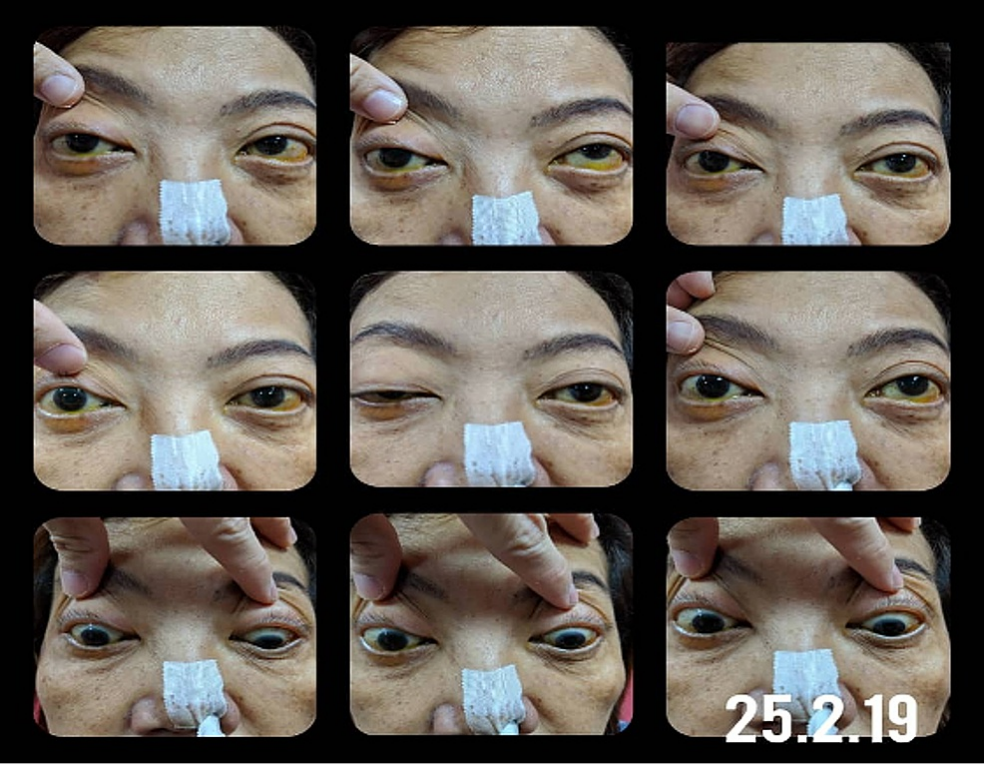
Total limitation of right eye extra-ocular movements in all directions of gaze. Moderate limitation of left eye extraocular movements in all directions of gaze.

She was subsequently lost to follow up, but presented a year later with right eye ptosis for two weeks, associated with progressive worsening of diplopia and blurring of vision. On examination, the right eye was proptosed, with complete ptosis and complete ophthalmoplegia. The left eye had moderate limitation of extraocular movements in all directions of gaze (Figure 1). Right eye visual acuity (VA) was hand movements, with a right eye relative afferent pupillary defect, while the left eye VA was 6/9. Anterior segments, intraocular pressure and fundi were normal bilaterally. She was unable to read the Ishihara chart with her right eye. The left eye was able to read all 15 plates. Her right eye Humphrey Visual Field test showed a severely depressed visual field. However, the test was not reliable due to poor vision. Her left eye Humphrey Visual Field test also showed insignificant findings. Systemic examination revealed enlarged cervical lymph nodes. However, there was no cervical lymph node biopsy proceeded. 

**Figure 2:**
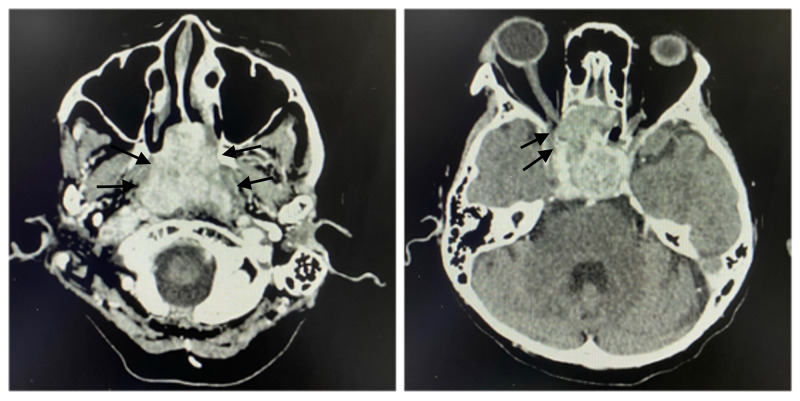
CT-scan shows a heterogenous enhancing soft tissue mass within nasopharynx, obliterating the fossa of Rosenmuller and torus tobarius bilaterally, with infiltration of skull base and right cavernous sinus.

**Figure 3:**
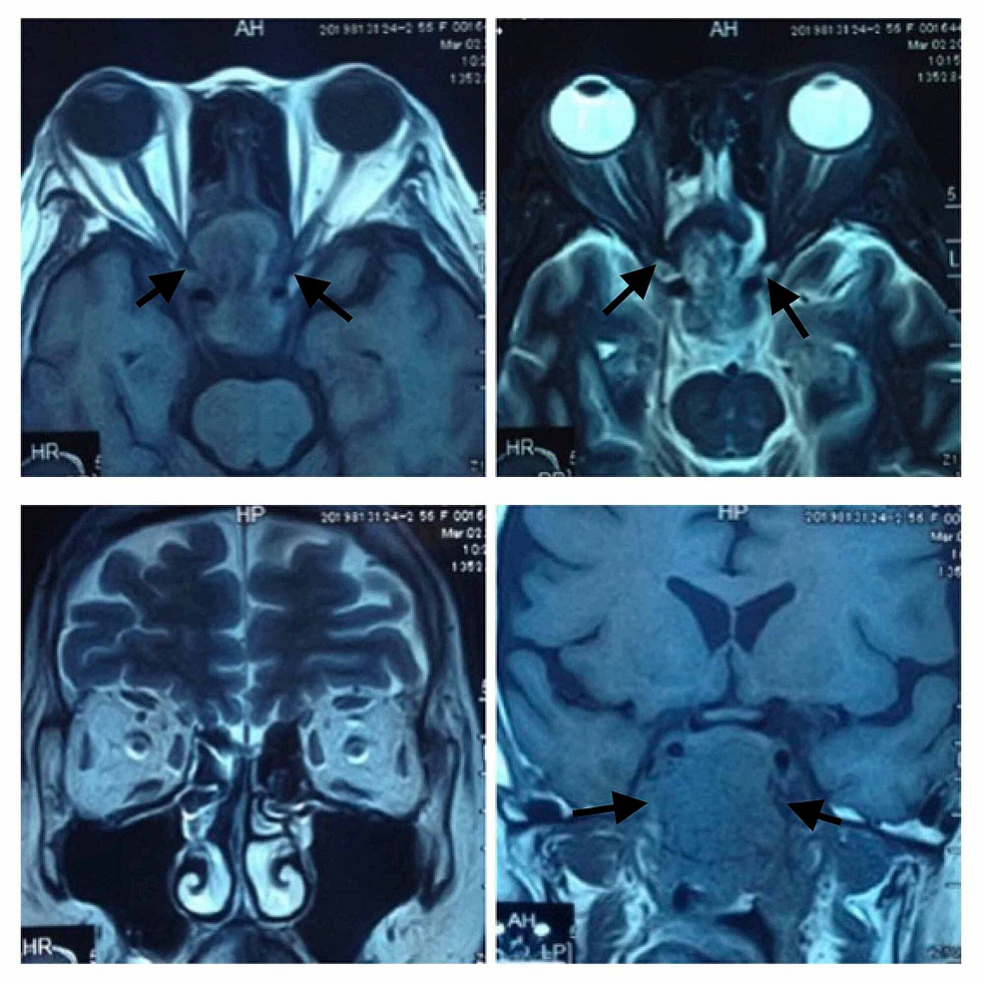
MRI shows extension of mass into orbital apex and bilateral optic canals. Mass extends superiorly to the midline of the optic chiasm and laterally into both cavernous sinuses, pushing the internal carotid vessels laterally.

Computed tomography scan (CT scan) showed a heterogenous enhancing soft tissue mass within the nasopharynx, obliterating fossa of Rosenmuller and torus tobarius bilaterally, with infiltration of the skull base and the right cavernous sinus (Figure 2). Magnetic resonance imaging (MRI) showed extension of the mass into the orbital apex and both optic canals, with greater involvement of the right side (Figure 3). The mass extended superiorly, touching the midline of the optic chiasm. The mass also extended laterally into both cavernous sinuses, pushing the internal carotid vessels laterally. 

The patient was started on intravenous methylprednisolone 250 mg four times a day for three days. Over this time, VA improved to 6/36 in the right eye, while the left eye vision was maintained at 6/9. Motility of both eyes also improved significantly. She was subsequently switched to oral prednisolone 1 g/kg/day, which was tapered over a period of seven months until she completed her concurrent chemoradiotherapy. This consisted of two cycles of cisplatin and fluorouracil, as well as radiotherapy 70 Gy/35#. By the end of this period, her vision was 6/9 in the right eye and 6/6 in the left, with normal colour vision and pupil responses bilaterally. The right eye motility had minimal residual limitation of abduction, while the left eye motility had normalized. Precautions and monitoring of corticosteroid side effects such as blood sugar level, blood pressure, risk of gastrointestinal bleeding and risk of secondary infection were taken into action throughout the treatment process. Patient was very fortunate as she did not experience any of these side effects. 

Unfortunately, four months later, her condition worsened, with complete loss of right eye vision, associated with poor oral intake, lethargy, cachexia and loss of weight. Ocular motility showed bilateral limited abduction and right eye VA of no light perception. CT scan showed progressive disease with enlargement of the previously noted nasopharyngeal mass and metastases to the liver, lymph nodes, adrenal glands and bones. She was restarted on another cycle of chemoradiotherapy.

## Discussion

NPC is most prevalent among the Chinese population, particularly for the age group of 40-49 years. Malaysia, being a multi-racial country, has one of the highest rates of NPC in Southeast Asia [5]. NPC commonly presents with pain such as headache and earache, nasal bleeding, nasal obstruction or stuffiness, hemoptysis and runny nose [6].

Cranial nerve involvement, commonly of the abducens and trigeminal nerves, occurs in approximately one-third of cases [2,4]. This is attributed to tumor invasion to the base of the skull and the cavernous sinus, as these two nerves are located at the floor of the cavernous sinus [2,4]. Optic nerve involvement rarely occurs until the disease has reached an advanced stage and tends to be associated with extraocular muscle involvement [7]. This is in keeping with our case, where the tumor invaded the right cavernous sinus, base of the skull and orbital apices, causing optic nerve involvement associated with multiple cranial nerve palsies. 

The main investigations in NPC are nasal endoscopy to identify the presence of a mass, followed by cross-sectional imaging such as CT scan, MRI or positron emission tomography (PET) for TNM staging [8]. MRI is more sensitive than CT scan in detecting local disease extension. MRI also is more sensitive in evaluating soft tissue changes such as infiltration of cranial nerves. However, PET is more sensitive and accurate than MRI in detecting distant metastasis. Biopsy provides the histological diagnosis required to plan treatment [8]. The choices of treatment are based on the results of radiological and histological studies. Radiotherapy is the treatment of choice for NPC and its regional nodal metastases. Chemotherapy is used as an adjunct for more advanced tumors. Surgery is mainly for biopsy [8]. 

Pulsed dose corticosteroid therapy has been reported to relieve optic neuropathy, both due to inflammatory and compressive causes. Even in cases of paraneoplastic optic neuropathy, corticosteroids may still be beneficial via their anti-inflammatory effects. In NPC patients treated with a corticosteroid regime during chemoradiotherapy, additional improvement of vision has been noted after the aforementioned therapy [9]. Some authors hypothesize that the dramatic visual improvement in compressive optic neuropathy after treatment with methylprednisolone is due to reduction in tissue oedema, thus reducing the compressive effects of the tumour. This buys time while awaiting the debulking effect of chemo-radiotherapy [10]. Prolonged maintenance of oral steroids while undergoing chemoradiotherapy may provide an additional effect in reducing the compressive effects of the tumour. In our case, IV methylprednisolone resulted in immediate visual improvement, which was maintained with oral steroids throughout the chemoradiotherapy. This corticosteroid therapy also did not compromise the primary therapeutic regime of chemoradiotherapy, either in its efficacy or toxicity. 

## Conclusions

Involvement of the optic nerve in NPC is a sign of advanced disease. Corticosteroids are beneficial in preserving vision in optic neuropathy secondary to NPC.
